# N-Acetylcysteine in Schizophrenia: A Convergent Redox-Based Approach to Therapeutic Potential

**DOI:** 10.3390/antiox15101277

**Published:** 2026-10-02

**Authors:** Enric Rubio-Contreras, Alonso Pérez-Toribio, Patricia Álvaro, Ana M. Cosialls, Constanza Morén

**Affiliations:** 1 August Pi i Sunyer Biomedical Research Institute (IDIBAPS), 08036 Barcelona, Spain; enrubio@recerca.clinic.cat; 2 Clínic Foundation for Biomedical Research (FCRB), 08036 Barcelona, Spain; 3 Department of Fundamental and Clinical Nursing, Faculty of Nursing, University of Barcelona, 08907 Barcelona, Spain; anamcosialls@ub.edu; 4 CSMA Dreta de l’Eixample, Fundació CPB Serveis Salut Mental, 08025 Barcelona, Spain; palvaro@cpbssm.org

**Keywords:** antioxidant, glutamatergic dysfunction, glutathione, N-acetylcysteine, NMDAR hypofunction, oxidative Stress, parvalbumin interneurons, schizophrenia

## Abstract

Schizophrenia (SCZ) remains one of the most disabling psychiatric disorders, in large part because current antipsychotics (APs) inadequately address negative symptoms and cognitive deficits. Converging evidence implicates a set of interconnected mechanisms, NMDAR hypofunction, oxidative stress, and a possible low-grade neuroimmune activation, that converge on parvalbumin-positive GABAergic interneuron dysfunction, the likely substrate of these refractory symptom domains. N-Acetylcysteine (NAC) is an adjunctive candidate because it may act on several of these pathways: it replenishes glutathione (GSH), restores redox balance, and modulates glutamatergic transmission, while its immunomodulatory effects remain poorly characterized in SCZ. This comprehensive review synthesizes evidence from preclinical models and clinical trials, showing that NAC produces replicated but modest benefits for negative symptoms and cognitive function, with inconsistent effects on positive symptoms. Notably, null findings in clinical-high-risk and treatment-resistant populations, together with a single exploratory subgroup analysis in early psychosis in which a peripheral redox index (erythrocyte glutathione peroxidase activity) was associated with positive-symptom response, raise the hypothesis that NAC efficacy may depend on a pre-existing, measurable antioxidant deficit. Adequately powered, biomarker-enriched trials that stratify patients by redox status are therefore needed to determine whether NAC is an effective targeted therapy for a mechanistically defined SCZ subgroup. Such trials would resolve whether the modest effect sizes observed in unselected populations reflect true but limited efficacy or dilution by enrolment of redox-normal patients unlikely to benefit.

## 1. Introduction

Current antipsychotic (AP) treatments for schizophrenia (SCZ) remain largely refractory to negative symptoms and cognitive impairments, with benefits primarily confined to positive symptoms [1]. Mechanistically, positive symptomatology is associated with dopaminergic metabolism [2], whereas negative and cognitive deficits are more closely linked to glutamatergic metabolism [3]. These limitations reflect, at least in part, the clinical heterogeneity and severity of SCZ, underscoring the need for innovative therapeutic strategies [4,5].

Etiopathogenesis of SCZ has long been conceptualized within the dopamine hypothesis, which has remained the dominant explanatory model for more than 50 years [4,5,6]. Despite decades of research, therapeutic advances have been modest, highlighting the need to explore novel pathophysiological mechanisms and treatment strategies beyond dopaminergic modulation [4,7]. Converging lines of evidence point to impairments in both serotonergic and glutamatergic systems, supporting their role as key contributors to the varied pathophysiological mechanisms in SCZ [8,9,10]. Specifically, glutamatergic dysfunction has emerged as a central mechanistic framework, centered on the N-methyl-d-aspartate receptor (NMDAR) hypofunction hypothesis in parvalbumin-positive GABAergic interneurons (PVIs). This hypothesis stems from findings that NMDAR antagonists like phencyclidine and ketamine induce an SCZ-like state in healthy individuals, producing not only positive symptoms, but also negative symptoms and cognitive deficits [11,12]. This pharmacological model has been supported by both rodent [13] and human studies [14], demonstrating increased glutamate levels in the prefrontal cortex (PFC) and reduced dendritic complexity [12]. Moreover, oxidative stress has been identified as an additional interconnected pathway contributing to glutamatergic dysfunction via PVIs [15]. This is characterized by increased oxidative damage and reduced capacity of intracellular redox modulatory systems [16], which may contribute to direct impairment of NMDAR function [17]. Therefore, a growing body of evidence suggests that these alterations have a profound impact on PVIs, which are particularly vulnerable to oxidative stress [18] due to their high metabolic demands and low antioxidant capacity [15], ultimately affecting the generation of gamma oscillations underlying cognitive and perceptual processes [19].

**Figure 1:**
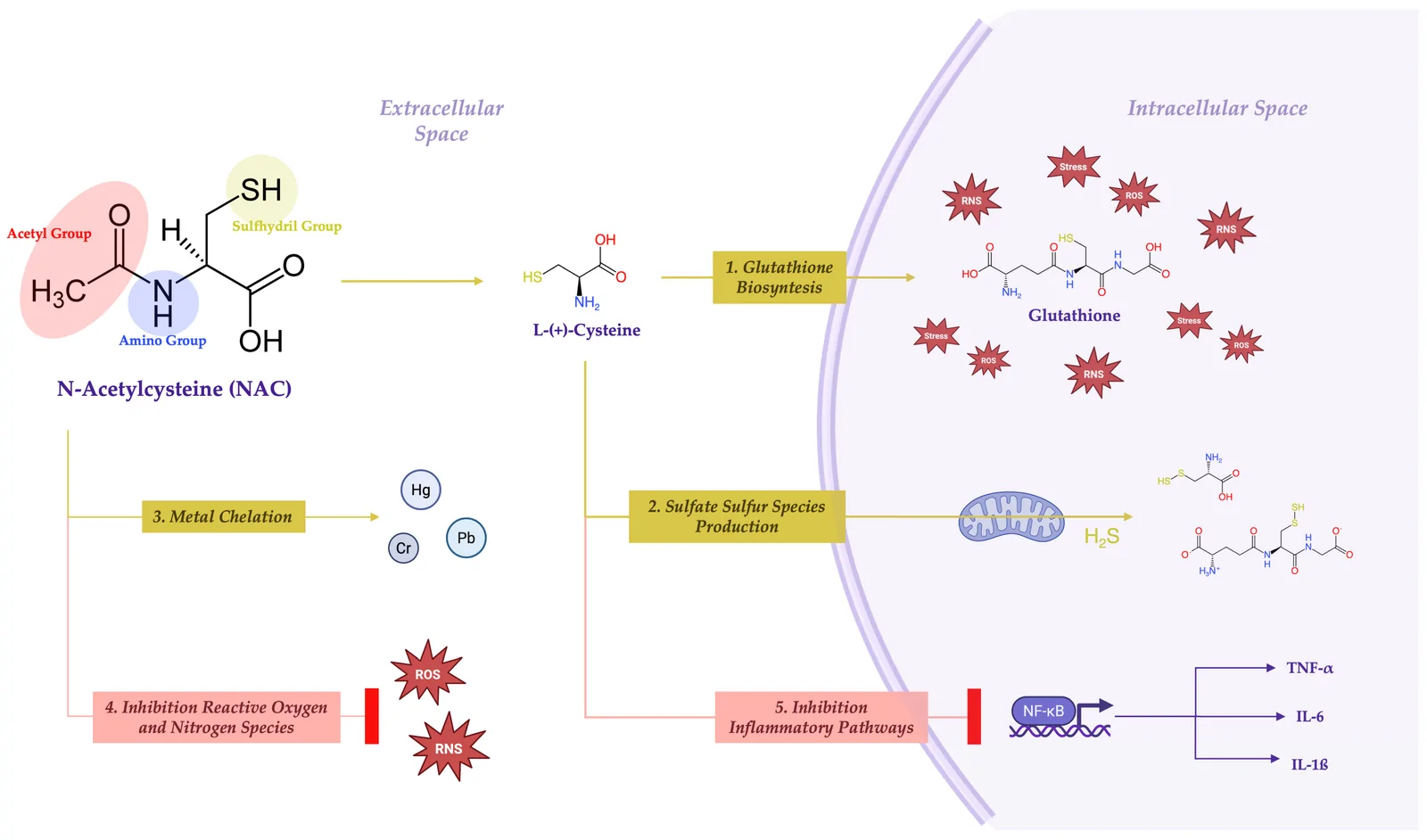
N-Acetylcysteine: Mechanisms of Action in Redox Modulation, Glutathione Biosynthesis, and Anti-inflammatory Effects. NAC features an acetyl group (CH3CO-) attached to the amino nitrogen (-NH-); however, the key structural element, responsible for most of its biological activity, relies on the free sulfhydryl group (-SH) attached to the cysteine part of the molecule. This molecule shows direct antioxidant efficacy by: (1.) acting as a precursor of L-cysteine and, consequently, of GSH to replenish the intracellular GSH pool in conditions of oxidative stress, (2.) increasing sulfate sulfur species production inside cells, which provides additional protective effects independently of GSH replenishment, (3.) combining its sulfhydryl portion to redox active metal ions, forming complexes that are easily excreted by the body, (4.) interacting through its thiol group with RONS, such as NO_2_ and •OH, and (5.) inactivating the NF-κB, which plays a critical role in the inflammatory cascade and immune response. Abbreviations: Cr: Chromium, Hg: Mercury, IL-1ß: Interleukin-1 beta, IL-6: Interleukin-6, NAC: N-Acetylcysteine, NF-κB: Nuclear Factor Kappa-light-chain-enhancer of activated B cells, Pb: Lead, RNS: Reactive Nitrogen Species, ROS: Reactive Oxygen Species, TNF-α: Tumor Necrosis Factor-Alpha. While NAC also displays biochemical effects beyond redox-related mechanisms, including the reduction of disulfide bonds, the present figure highlights its most relevant functions linked to redox regulation.

The redox system, particularly glutathione (GSH), is associated with glutamate function, and vice versa. GSH is synthesized in the cytosol of all mammalian cells in a tightly regulated manner. The major determinants of GSH synthesis are the availability of cysteine, the sulfur amino acid precursor, and the activity of the rate-limiting enzyme, glutamate cysteine ligase [20]. Hence, given the interplay between redox imbalance and glutamatergic dysfunction, antioxidant therapy has gained attention as a potential strategy to target core mechanisms underlying SCZ [21,22]. Within this framework, several antioxidant compounds have been investigated as potential adjunctive treatments, such as omega-3 fatty acids [23], vitamin C [24], and sulforaphane [25], among others [26,27]. However, clinical findings across this class remain heterogeneous and often inconsistent, which limits confidence in any broad therapeutic effect [22,28]. This variability highlights the importance of targeting specific pathophysiological pathways, as reflected in the investigation of compounds with defined mechanisms such as omega-3 fatty acids [29,30] and novel negative-symptom interventions [31]. N-Acetylcysteine (NAC) represents one such candidate, having been evaluated in several clinical trials that demonstrated modest efficacy in ameliorating negative symptoms and cognitive deficits [32], both inadequately addressed by AP [1]. Nevertheless, findings are not entirely consistent across studies regarding positive symptoms [33]. These beneficial interactions, particularly in negative and cognitive symptoms, likely result from NAC’s chemical structure and function (Figure 1), acting primarily as a direct and indirect antioxidant through GSH synthesis [34,35], and restoration of redox balance [36,37,38]. Moreover, NAC has been shown to modulate glutamatergic neurotransmission [39] and exert neuroimmune modulatory effects, thereby acting at the intersection of multiple biological systems implicated in SCZ [34]. Collectively, current findings indicate that NAC may represent a promising therapeutic strategy, particularly when redox imbalance is present [40]. However, further research is needed, as the available clinical data suggest only modest overall benefit and possible value in selected subgroups [34,39] and symptoms [33].

This narrative review aims to explore the therapeutic potential of NAC in SCZ. Specifically, it examines its effects on glutamatergic signaling and related NMDAR hypofunction, oxidative stress, and low-grade neuroimmune activation, as well as its efficacy in improving negative symptoms and cognitive deficits.

### Search Strategy

A structured but non-systematic literature search was conducted, aimed at identifying the most relevant evidence on NAC as an adjunctive treatment in SCZ, with particular attention to glutamate dysfunction, oxidative stress, and their interaction in the pathophysiology of the disorder. Searches were conducted in PubMed, Scopus, and Web of Science from database inception to May 2026, using combinations of the terms «N-Acetylcysteine, schizophrenia, psychosis, negative symptoms, cognition, oxidative stress, glutathione, glutamatergic dysfunction, NMDAR hypofunction, PVIs, neuroinflammation, and redox imbalance». Particular focus was placed on original research, clinical trials, meta-analyses, and high-quality preclinical and translational studies that addressed the mechanistic rationale or therapeutic effects of NAC in SCZ. Studies were included if they provided information relevant to glutamatergic dysfunction, oxidative stress, redox imbalance, NAC mechanisms of action, or clinical outcomes such as negative symptoms, cognitive performance, or overall symptom burden. Articles were excluded if they were unrelated to SCZ, lacked primary or clearly relevant secondary evidence, or provided insufficient methodological detail.

## 2. NMDAR Hypofunction and Glutamate Impairments in SCZ

The NMDAR hypofunction hypothesis is supported by the finding that NMDAR antagonists induce an SCZ-like state in healthy individuals [11,12]. NMDAR hypofunction also underscores the need to consider mechanisms beyond dopamine dysfunction, given the limited effects of APs on negative symptoms and cognitive deficits [41].

This hypothesis centers on the NMDAR, one of the main ionotropic glutamate receptors expressed in PVIs [41], a class of fast-spiking GABAergic interneurons that regulate excitatory neuronal signaling. Suppression of these inhibitory neurons leads to disinhibition of excitatory neurons, which may contribute to the clinical manifestations of the disease [42]. Moreover, PVIs are among the most abundant cortical interneuron populations and have electrophysiological properties associated with high metabolic demand [18,43]. These cells are responsible for synchronizing the firing of pyramidal neurons, primarily in the PFC and hippocampus, thereby supporting the generation of gamma-band oscillations (30–100 Hz) critical to optimal cognitive function [41,44]. These oscillations contribute to acute synaptic plasticity and longer-term structural adaptations [41,45]. Because PVIs mature extensively during adolescence and do not reach full maturity until adulthood, this developmental period may represent a window of increased vulnerability to adverse insults [46]. In this context, stressors or preexisting abnormalities during adolescence may have particularly deleterious effects on PVI development and function [42,47]. Recent preclinical evidence further supports this developmental vulnerability, showing that NAC administered during or after adolescent stress attenuated subsequent behavioral alterations, oxidative damage, and PVI deficits in adult rats [48].

In this framework, NMDAR hypofunction reduces inhibitory control over pyramidal neuron activity [11,49]. Reduced cortical PVI labeling may reflect a loss of parvalbumin expression rather than frank interneuron death [47]. As a result, cortical neurons become disinhibited, leading to excessive glutamatergic output [9], and a paradoxical state of cortical hyperexcitability with disrupted excitation–inhibition balance [50]. Additionally, pyramidal neurons project to the ventral tegmental area via glutamatergic pathways, thereby contributing to subcortical hyperdopaminergia [44], which has been associated with positive symptoms in SCZ [3]. In light of these observations, dysfunction of cortical PVIs has been proposed as a contributing factor in neurodevelopmental disorders, such as SCZ [18]. Together, these findings are consistent with the gamma-frequency synchrony disturbances observed in SCZ [50], which contribute to working memory deficits [51], poorer performance on cognitive tasks [52], and impaired daily functioning [51].

These findings have led to the prevailing view that PVIs are dysfunctional in SCZ, although the causes and mechanisms remain incompletely understood [53]. An emerging hypothesis proposes the involvement of oxidative damage [47,54] and neuroimmune alterations during development, which could contribute to interneuron dysfunction and, thereby, PVI impairment [55].

## 3. Oxidative Stress and Altered Neuroimmune Activity in SCZ: Neuronal Vulnerability

Oxidative stress, resulting from aberrant redox control, has emerged as an attractive hypothesis for explaining, at least in part, the pathophysiology of SCZ [56,57], and the mechanisms associated with NMDAR dysfunction during early postnatal development [15,58]. Increased reactive oxygen species (ROS) production, when not adequately neutralized by antioxidant defenses [59], may have detrimental effects on macromolecules, ultimately altering gene expression [56]. Conversely, GSH helps maintain cellular redox homeostasis by scavenging reactive species and supporting antioxidant and detoxification pathways, thereby limiting oxidative damage [16].

During early adolescence, PVIs are highly plastic in terms of excitatory drive and neuronal activity [17,60]. Their susceptibility to oxidative stress in early development may have lasting consequences in adulthood [17]. This vulnerability is supported by experimental evidence from *Gclm* knockout mice, which exhibit a 60–70% reduction in brain glutathione levels. In this model, PVIs show increased susceptibility to oxidative stress, an effect that can be prevented by NAC administration [61]. Neurons also have relatively weak antioxidant protection, demonstrated by lower catalase content and reduced GSH levels compared with hepatocytes [62]. In addition, neurons contain substantial amounts of redox-active metals, such as iron, zinc, copper, and manganese, which can catalyze ROS formation [56]. Therefore, a dysregulated stress response may lay the foundation for impaired neuronal metabolism [15,57] and increased ROS generation, thereby contributing to oxidative stress and cellular damage [17]. Collectively, these findings indicate that disruption of NMDAR function during postnatal development may affect the normal maturation of PVIs through oxidative-stress-related mechanisms, perpetuating vicious feed-forward mechanisms [15,17,63], and modulating synaptic activity and plasticity [62]. Moreover, this imbalance may trigger the formation of oxidative radicals that can induce dendritic atrophy through local apoptosis, leading to some of the structural and functional alterations that are seen in psychosis [62]. Consequently, cognitive dysfunction has been associated with an imbalance in the generation and clearance of ROS and reactive nitrogen species [57]. Beyond PVIs, redox imbalance may also affect other vulnerable neural compartments, including oligodendrocytes and white matter, which are essential for efficient circuit connectivity [64].

Oxidative stress may further amplify SCZ-related pathology by engaging neuroimmune pathways, particularly through apoptotic activation and downstream cytokine dysregulation [58,65]. At a molecular level, oxidative stress can activate the nuclear factor kappa light-chain-enhancer of activated B cells (NF-κB), a rapid-acting transcriptional activator of inflammation responsible for promoting free-radical production [58,66], abnormal cytokine profiles [65], and altered IL-6, IL-8, and TNF-α levels [67], thereby contributing to apoptotic mechanisms [16]. These changes have been associated with executive dysfunction and clinical SCZ symptomatology [44,65]. Notably, it has been demonstrated that neuroinflammation is an unfortunate term for SCZ [68]. This pathology may involve, at most, a very-low-grade inflammatory state, distinct from the classical epidemiological definition of inflammation. Nevertheless, features consistent with low-grade neuroinflammation in SCZ may reflect context-dependent neuroimmune mechanisms that are adaptive, maladaptive, or compensatory [68].

Supporting these interlinked hypotheses, multiple human studies have reported impairments in oxidative stress markers in both peripheral blood and central nervous system tissue in SCZ [16,59]. Moreover, rodent models have similarly shown that dysregulated ROS production reduces sociability, impairs cognition, decreases PVI labeling, and produces electrophysiological and transcriptional changes relevant to SCZ [60]. In individuals with SCZ, downregulation of antioxidant synthesis pathways and increased ROS production have been observed across multiple groups [17,64], consistent with reduced GSH levels in the PFC and post-mortem SCZ brains, where they correlate with more severe negative symptoms [17]. Overall, lower antioxidant levels and higher ROS values appear to be core features of the disorder and are not fully explained by disease progression or medication, supporting redox imbalance as a primary characteristic of SCZ [64]. Interestingly, executive dysfunction in SCZ also correlates with higher ROS levels and lower antioxidant-related protein levels, directly linking redox imbalance to cognitive impairment [65].

**Figure 2:**
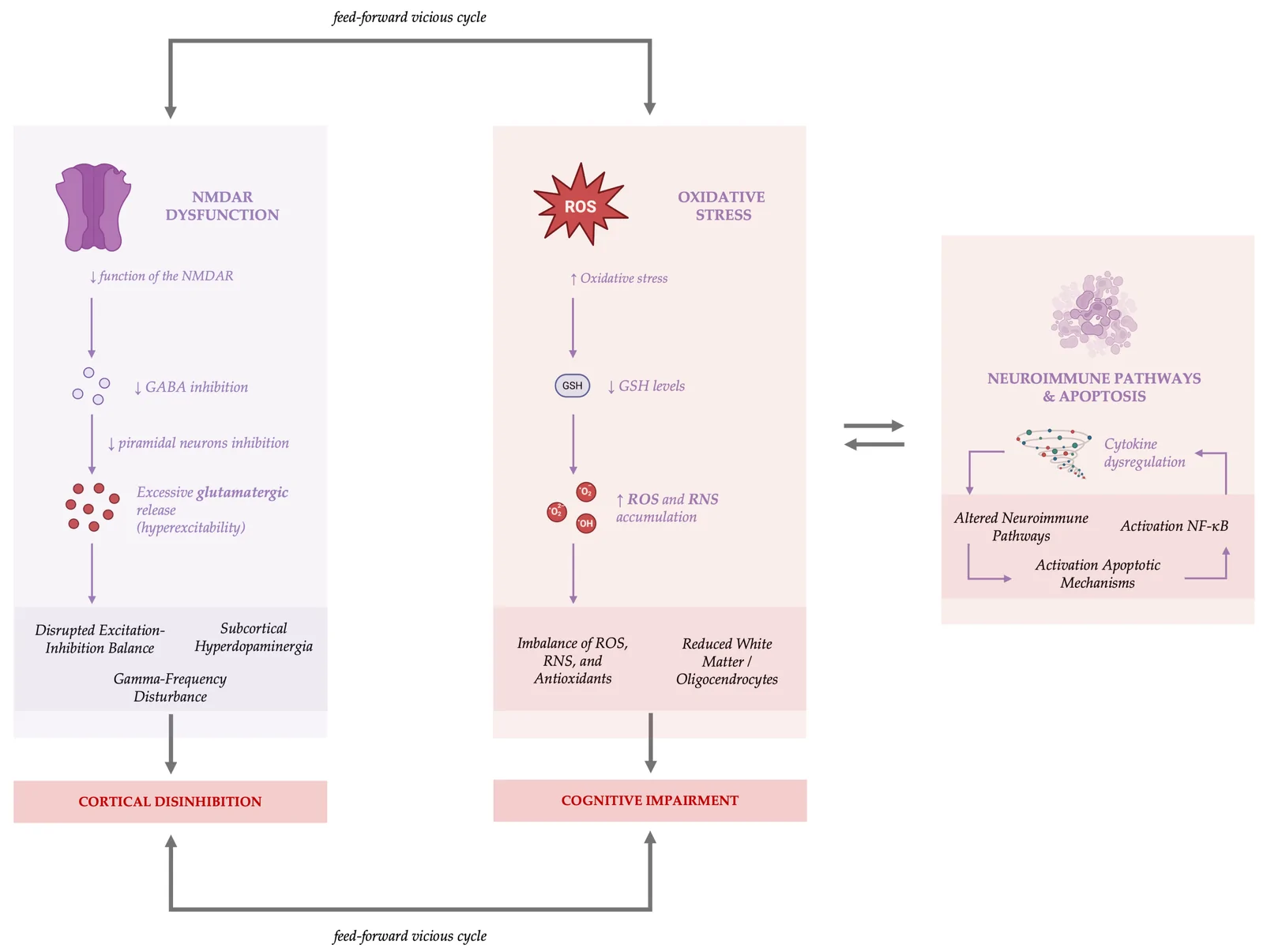
Integrative model of PVI dysfunction in SCZ. The schematic depicts a feed-forward pathological cascade in which NMDAR dysfunction in parvalbumin interneurons reduces GABAergic inhibitory tone, resulting in pyramidal neuron disinhibition, excessive glutamatergic release, disruption of the excitation–inhibition balance, and subcortical hyperdopaminergia. In parallel, oxidative stress, characterized by reduced GSH levels and accumulation of RONS, interacts with dysregulated neuroimmune pathways and apoptosis, further contributing to white matter and oligodendrocyte reduction, and ultimately cognitive impairment. Abbreviations: ↑ indicates an increase; ↓ indicates a decrease, GABA: Gamma-Aminobutyric Acid, GSH: Glutathione, NMDAR: N-methyl-D-aspartate Receptor, O_2^−^_: Superoxide, O_2_^2−^: Peroxide, RONS: Reactive Oxygen and Nitrogen Species, ROS: Reactive Oxygen Species. Neuroimmune activation and local apoptotic processes remain incompletely understood and are currently supported by limited evidence. For this reason, they are not extensively discussed in the present review; however, they have been included in the figure for illustrative purposes.

Therefore, current evidence suggests that oxidative stress and NMDAR hypofunction may contribute to cortical oxidative damage, GSH deficiency, and reduced antioxidant activity through transcriptional regulation [62]. Additionally, this intertwined pattern appears to be mechanistically interdependent rather than sequential, forming bidirectional, self-amplifying vicious circles in which either can initiate the pathological cascade and each can worsen the other through positive feed-forward loops [17] (Figure 2). However, although the exact molecular framework is not yet fully established, preliminary results suggest NAC antioxidant therapy as a potential intervention to improve cognitive and negative functioning in individuals with SCZ [32].

## 4. Implications of N-Acetylcysteine in the Glutamate Hypothesis and Redox Imbalance

Based on genetic studies [69], in vivo [70], and in vitro cellular models [71], NAC has been proposed as a potential therapeutic agent in SCZ. Its effects are primarily attributed to its role as a precursor of GSH, which in turn supports modulation of glutamatergic and dopaminergic systems, reduces oxidative stress, and stabilizes neuroimmune activity [72].

Consistent with these mechanisms, several clinical trials have evaluated NAC in SCZ (Table 1), reporting moderate benefits, particularly for negative symptoms [73,74], which are largely refractory to current APs [1,41]. Neurobiological studies further support these findings: compared with the placebo group, NAC treatment has been associated with increased GSH levels (*F* = 4.86, *p* = 0.035) and a trend toward reduced glutamate levels *(F* = 4.04, *p* = 0.054) after 8 weeks, both of which are implicated in SCZ pathophysiology [75]. In contrast, an acute magnetic resonance spectroscopy (MRS) challenge study found that a single 2400 mg dose of NAC produced no change in cortical GSH or glutamate levels in the dACC or mPFC of patients with SCZ [76], consistent with the possibility that central target engagement by NAC may require sustained, long-term administration rather than acute dosing to restore redox balance. In addition, neuroimaging studies using magnetic resonance imaging have provided preliminary evidence that NAC modulates functional connectivity within brain networks relevant to SCZ, including reduced resting-state connectivity in PFC regions [77]. These neurochemical and circuit-level changes may underlie observed functional enhancements [78]. Converging preclinical and clinical findings reinforce this model: in *Gclm* knockout mice, PVIs are highly vulnerable to oxidative stress due to GSH deficiency, whereas in patients, NAC administration increases prefrontal GABA levels and improves cognitive function, suggesting restoration of inhibitory circuitry through redox modulation [79].

Clinically, longer-term NAC administration has been associated with significant improvements in symptom severity [78]. A 52-week study reported reductions in PANSS total (*F* = 14.7, *p* < 0.001), negative (*F* = 5.1, *p* = 0.024), and disorganized thought factor scores (*F* = 13.7, *p* < 0.001) [78]. Similarly, NAC at 2000 mg/day significantly improved PANSS negative (p = 0.018), general (*p* = 0.035), and total (*p* = 0.009) scores at week 24 compared with baseline [80]. NAC may also impact cognitive function, particularly in individuals with more chronic illness [81]. Improvements in working memory were observed after 24 weeks of treatment (*U* = 98.5, *p* = 0.027) [81]. Moreover, in a single early psychosis trial (2700 mg/day, 6 months), three separate analyses of the same cohort each reported a distinct outcome: NAC was associated with functional connectivity [82], reflected in verbal fluency (*F* = 5.75, *p* = 0.023), trail-making tests (*F* = 4.28, *p* = 0.048), processing speed (*F* = 5.85, *p* = 0.022) [40], and, for the first time, with enhanced white matter integrity after treatment [83]. In the same trial, a peripheral redox index based on blood cells’ glutathione peroxidase activity identified a subgroup of early-psychosis patients in whom positive symptoms improved with NAC [40]. Other studies have also reported reductions in positive symptomatic scores (*F* = 5.47, *df* = 1, *p* = 0.02), along with improvements in negative symptoms, general psychopathology, and total score compared with placebo [33]. In individuals at high risk of psychosis, NAC showed a non-significant trend toward reduced transition risk [84], alongside improvements in prodromal symptoms [85]. Additionally, preliminary results from a 2400 mg/day NAC administered for 8 weeks found increased neural synchronization in SCZ [86], a disturbance that has been linked to NMDAR hypofunction [87]. Consistent with this, individuals with SCZ also showed improved auditory cortical functioning (*p* = 0.025) [88].

Despite these promising findings, results remain heterogeneous, and some studies show no benefit in specific clinical conditions [40,75,88,89]. A consistent observation is that positive symptomatology may not substantially benefit from NAC treatment [74,78], as many trials have not demonstrated significant improvements in PANSS positive scores [74,78], although findings are not entirely consistent across studies [33]. Additionally, NAC has not demonstrated efficacy in treatment-resistant populations [90], and molecular findings regarding GSH and glutamate levels are, to a small extent, heterogeneous [76]. Furthermore, the immunological effects of NAC remain largely unexplored, as no clinical trials have specifically assessed immune responses and apoptosis to treatment in SCZ.

Therefore, adequately powered, real-world studies with longer follow-up periods are needed to clarify the clinical role of NAC and to identify subgroups most likely to benefit. While NAC is generally considered safe and well-tolerated [33], some adverse events have been reported, most commonly involving the gastrointestinal system, although these have not been deemed clinically significant [91]. In this context, NAC remains a candidate treatment whose clinical role would be best clarified by adequately powered, biomarker-informed trials.

**Table 1:** Studies addressing NAC treatment in people living with SCZ, Schizoaffective Disorder, or high-risk populations.

Author, Year	Sample	NAC Dose	Time Duration	Results
Farokhnia, M. et al. (2013) [74]	**·** 42 participants diagnosed with SCZ: - NAC (*n* = 21) - Placebo (*n* = 21)	2000 mg/day	8 weeks	NAC augmentation was associated with significantly greater improvement in PANSS total and negative subscale scores than placebo, while differences were not significant for positive symptoms, general psychopathology, or adverse effects.
Yang, Y. et al. (2022) [75]	**·** 34 participants diagnosed with SCZ and SZA: - NAC (*n* = 18) - Placebo (*n* = 16)	2400 mg/day	8 weeks	NAC was associated with increased medial PFC GSH and a trend toward reduced glutamate relative to placebo, whereas no significant treatment effects were observed for cognition, negative symptoms, dorsolateral PFC measures, or peripheral GSH indices.
McQueen, G. et al. (2020) [77]	**·** 17 participants diagnosed with SCZ: - NAC or Placebo (*n* = 17)	2400 mg/two doses × 7 days apart	1 week	NAC was associated with significantly reduced resting-state functional connectivity relative to placebo within the default mode and salience networks, especially between the medial PFC and mid frontal gyrus, and between the anterior cingulate cortex and frontal pole, with all reported effects at *p* < 0.04.
Breier, A. et al. (2018) [78]	**·** 60 participants diagnosed with SSD: - NAC (*n* = 30) - Placebo (*n* = 30)	3600 mg/day	52 weeks	NAC was associated with significant improvement versus placebo in PANSS total, negative symptom, and disorganized thought scores, but it did not improve positive symptoms or BACS cognitive scores. In preliminary MRI analyses, NAC did not produce significant changes in brain morphology over the treatment period.
Berk, M. et al. (2008) [80]	**·** 140 participants diagnosed with SCZ: - NAC (*n* = 69) - Placebo (*n* = 71)	2000 mg/day	28 weeks	NAC was associated with clinically meaningful benefit relative to placebo, including significantly greater improvement on CGI-S and on PANSS negative, general, and total scores at week 24, with the clearest separation emerging after longer exposure rather than at week 8.
Rapado-Castro, M. et al. (2017) [81]	**·** 58 participants diagnosed with SCZ/BP: - NAC (*n* = 27) - Placebo (*n* = 31)	2000 mg/day	24 weeks	At 24 weeks, participants receiving NAC showed significantly better working memory performance than placebo on digit span backwards, with the between-group comparison reaching significance, but no significant differences were found for the other cognitive domains assessed.
Conus, P. et al. (2018) [40]	**·** 61 participants diagnosed with early psychosis: - NAC (*n* = 31) - Placebo (*n* = 30)	2700 mg/day	26 weeks	No significant between-group benefit of NAC on negative symptoms or functional outcomes was found, but it reported improvement in processing speed and increases in glutathione-related biomarkers, suggesting a biomarker-associated signal rather than broad clinical efficacy.
Mullier, E. et al. (2019) [82]	**·** 20 participants diagnosed with early psychosis: - NAC (*n* = 9) or Placebo (*n* = 11) **·** 74 healthy controls	2700 mg/day	26 weeks	Functional connectivity along the cingulum bundle was increased after 6-month NAC supplementation in early psychosis patients. However, no significant differences in functional connectivity were found between the 2 groups.
Klauser, P. et al. (2018) [83]	**·** 20 participants diagnosed with early psychosis: - NAC (*n* = 10) - Placebo (*n* = 10)	2700 mg/day	26 weeks	For the first time, the administration of NAC, increases white matter integrity in the fornix. These results suggest that a GSH increase through NAC supplementation may improve/protect white matter integrity, at least in the fornix.
Sepehrmanesh, Z. et al. (2018) [33]	**·** 79 participants diagnosed with SCZ: - NAC (*n* = 40) - Placebo (*n* = 39)	1200 mg/day	12 weeks	NAC was associated with significant improvement in positive and negative PANSS symptoms, with general and total PANSS scores decreasing over time relative to placebo, and there were also signals of improvement in selected cognitive domains, including attention, short-term and working memory, executive function, and processing speed; adverse effects did not differ significantly between groups.
Wasserthal, S. et al. (2024) [84]	**·** 48 individuals with clinical high risk for psychosis: - NAC (*n* = 23) - Placebo (*n* = 25)	2000 mg/day	26 weeks	The factorial 2 × 2 trial did not show a statistically significant advantage of NAC over placebo for preventing transition to psychosis or symptom deterioration, although NAC groups showed numerically higher event-free survival and lower transition rates than placebo groups.
Miyake, n. et al. (2016) [85]	**·** 5 participants presenting with prodromal symptoms: - NAC (*n* = 5)	2000 mg/day	12 weeks	NAC demonstrated a tendency to reduction in the Scale of Prodromal Symptoms total score from baseline to endpoint. Moreover, significant improvements were observed in the Brief Assessment of Cognition in SCZ composite z score (*p* < 0.043) and the SCZ Cognition Rating Scale score (*p* < 0.038).
Yang, Y. et al. (2019) [86]	**·** 19 participants diagnosed with SCZ: - NAC (*n* = 9) - Placebo (*n* = 10)	2400 mg/day	8 weeks	The study demonstrated an NAC-associated increase in neural synchronization assessed with auditory steady-state response in SCZ. Total power at 40 Hz stimulation was improved with NAC.
Lavoie, S. et al. (2008) [88]	**·** 7 participants diagnosed with SCZ: - NAC and Placebo (*n* = 7)	2000 mg/day	17 weeks	Administration of NAC, to SCZ patients resulted in improved auditory cortical functioning. No increase in cysteine levels was observed in blood from patients after NAC treatment.
Soheilipour, A. et al. (2025) [89]	**·** 59 participants diagnosed with SCZ: - NAC (*n* = 29) - Placebo (*n* = 30)	600 mg/day	8 weeks	NAC treatment resulted in a significant improvement in cognitive and positive indices, as well as an increase in peripheral GSH levels (*p* < 0.05). However, no significant impact was found in negative symptoms improvement.
Neill, E. et al. (2022) [90]	**·** 85 participants diagnosed with SCZ and SZA: - NAC (*n* = 42) - Placebo (*n* = 43)	2000 mg/day	52 weeks	NAC did not significantly improve negative symptoms, cognition, or quality of life relative to placebo over the 1-year treatment period, and side-effect burden did not differ between groups. An exploratory analysis suggested a small improvement in depressive symptoms.
Girgis, R. et al. (2019) [76]	**·** 19 participants diagnosed with SCZ and SZA: - NAC (*n* = 19) **·** 20 healthy controls	2400 mg/once	60–120 min	No differences in mPFC or dACC Glu levels or in dACC GSH levels were observed between patients with SCZ and healthy control subjects either at baseline or after one dose of NAC.

Given the comprehensive nature of this review, the studies included in this table are a representative but non-exhaustive selection of the available clinical trials on NAC in SCZ. Rows [40,82,83] report three separate analyses of the same early-psychosis randomized controlled trial (NAC 2700 mg/day, 6 months), each examining a different outcome (symptoms, neurocognition and redox biomarkers [40]; functional connectivity [82];: white-matter integrity [83]). They are not independent trials. Abbreviations: BACS: Brief Assessment of Cognition in Schizophrenia, CGI-S: Clinical Global Impressions-Severity, dACC: Dorsal Anterior Cingulate Cortex, GSH: Glutathione, mPFC: Medial Prefrontal Cortex, MRI: Magnetic Resonance Image, NAC: N-Acetylcysteine, PANSS: Positive and Negative Syndrome Scale, PFC: Prefrontal Cortex; SZA, schizoaffective Disorder; SCZ: Schizophrenia.

## 5. Discussion

This review synthesizes evidence that NAC targets a set of interconnected mechanisms, glutamatergic signaling and related NMDAR hypofunction [41], oxidative stress [54], and a possible low-grade neuroimmune activation [68], that converge on PVI dysfunction in SCZ. By potentially addressing these pathways simultaneously, NAC offers a mechanistic rationale that is distinct from dopaminergic APs, which do not directly target redox or glutamatergic systems [1].

Among antioxidant compounds investigated in SCZ, NAC has one of the strongest mechanistic rationales and the most replicated clinical evidence [33,34]. Several features distinguish it from agents such as omega-3 fatty acids [23], vitamin C [24], and sulforaphane [25], among others [26,27]. First, NAC is a precursor of GSH rather than a simple free-radical scavenger [92]. Second, it modulates glutamatergic transmission [77], thereby engaging two core pathophysiological pathways implicated in SCZ, redox imbalance and glutamate dysfunction, that other antioxidants address only partially or not at all [54]. Third, it crosses the blood–brain barrier and increases brain GSH levels, as confirmed by magnetic resonance spectrometry [75]. Finally, it has pragmatic advantages including oral bioavailability, a well-tolerated safety profile at doses up to 3600 mg/day, and low cost [78]. Consistent with this mechanistic profile, NAC has received a provisional recommendation for use in SCZ from the World Federation of Societies of Biological Psychiatry and the Canadian Network for Mood and Anxiety Treatments [93].

Results from several clinical trials demonstrate that negative symptoms and cognitive deficits appear to respond favorably to NAC treatment [78,81], whereas positive symptoms show modest and inconsistent improvement [33,74]. Although the overall literature suggests greater benefit for negative and cognitive symptoms, one trial reported a positive-symptom improvement confined to a post-hoc redox-defined subgroup [40], a single, as-yet-unreplicated finding. This positions NAC as one of the few adjunctive agents with replicated benefit for symptom domains that are largely refractory to current APs [1]. Importantly, all reviewed clinical trials administered NAC as an add-on to ongoing AP medication. This reinforces that NAC should be understood strictly as an adjunctive strategy: it adds value where APs fall short, rather than replacing them where they already work [94]. Its therapeutic value lies in covering the domains that APs leave undertreated [44], while they continue to manage positive symptoms effectively [95]. However, the evidence base remains limited. Results are not fully consistent across studies [40,75], and the factors that predict treatment response are not yet well understood. Not all studies report significant results associated with NAC treatment. Some trials have yielded negative findings for their primary endpoints. This heterogeneity in outcomes mirrors the broader controversy in the literature regarding NAC efficacy in SCZ [40,75,88,89]. Null findings can be explained by brief exposure to NAC (60–120 min), which may have been insufficient to produce detectable biological or clinical effects [76]. NAC did not either show benefit in clinical high-risk individuals [84] or in treatment-resistant populations [90]. One possible explanation, which we advance as a hypothesis rather than an established mechanism, is that its efficacy may depend on a specific redox state present in the early-to-mid stages of chronic SCZ but not yet established in at-risk individuals or exhausted in refractory illness. It is important to note that this stage-dependent account is, at present, derived largely from a single post-hoc subgroup analysis [40] and from the pattern of null results across populations; it has not been tested directly. The biomarker-stratified trial we propose below is intended precisely to test it, by measuring redox status prospectively rather than inferring it from illness stage. In the meantime, NAC may still be effective in a specific subpopulation of patients. Studies reporting negative results may have enrolled heterogeneous populations with varying redox status, which would increase within-group variability and dilute any signal that exists in a subgroup.

Heterogeneity across studies may partly reflect the fact that previous trials enrolled unselected SCZ populations. One promising way forward is biomarker-stratified enrichment [40]. In that trial (63 early-psychosis patients), NAC produced no significant effect on positive or negative symptoms in the sample as a whole; in an exploratory, post-hoc subgroup analysis, however, patients with high baseline erythrocyte glutathione peroxidase activity showed a link between change in redox status and improvement in positive symptoms, whereas the unstratified sample showed none. We emphasize that this is a single, unreplicated, hypothesis-generating result, not an established predictive effect. It is precisely this exploratory status that motivates the trial we propose: a prospective, biomarker-stratified design is the appropriate way to test whether the redox-defined subgroup signal is real, rather than to assume it [40]. Such a design has not yet been applied prospectively in chronic SCZ. We propose a multicenter, double-blind, randomized, placebo-controlled trial in which patients with stable SCZ, and at least moderate negative symptoms (PANSS negative ≥18), are screened for erythrocyte glutathione peroxidase activity, glutathione disulfide (GSSG) as an index of oxidative load, and total antioxidant capacity at baseline. Incorporating GSSG is supported by recent redox-metabolomic evidence that, in drug-naïve first-episode SCZ, GSSG is an independent correlate of cognitive impairment, whereas GSH and the GSH-to-GSSG ratio show weaker and less stable associations [96]; measuring the oxidized pool alongside antioxidant capacity would therefore index the redox imbalance more completely and is particularly relevant to the trial’s cognitive secondary endpoints. Only patients whose redox index falls below the screening population median, indicating a measurable antioxidant deficit, would be randomized to NAC 2400 mg/day or a matching placebo for 24 weeks. The primary endpoint would be the change in PANSS negative subscale score, a domain where NAC has shown replicated but modest benefit across prior trials at similar doses and durations [78,80,81]. Secondary endpoints would include working memory assessed by digit span backwards [81], processing speed [40], and resting-state functional connectivity within the default mode and salience networks [77], providing a bridge between peripheral biomarkers, brain-circuit outcomes, and clinically meaningful symptom improvement. This design would test two linked hypotheses: that NAC efficacy may be confined to patients with a pre-existing redox imbalance, and that the effect is largest for negative symptoms and cognition, precisely the domains where current APs are inadequate. An adequately powered, biomarker-enriched trial of this kind would either confirm NAC as a targeted therapy for a mechanistically defined subgroup or clarify that its modest effect size in unselected populations does not justify routine clinical use.

The findings reported here must be interpreted considering several methodological limitations that constrain the conclusions that can be drawn from the current evidence base. First, the sample sizes of the included clinical trials were small, ranging from 17 to 140 participants, which limits statistical power to detect moderate effect sizes and precludes robust subgroup analyses. Second, there was substantial heterogeneity across studies in NAC dosing (1200 to 3600 mg/day), treatment duration (60 min to 52 weeks), and baseline patient characteristics (early psychosis, chronic SCZ, treatment-resistant populations, and clinical high-risk states), making direct comparisons across trials difficult and potentially contributing to the inconsistency of findings across symptom domains. Third, most trials did not include prospective measurement of mechanistic biomarkers, such as erythrocyte glutathione peroxidase activity, plasma GSH levels, or in vivo brain GSH concentrations, making it impossible to confirm that NAC engaged its intended molecular target or to distinguish true non-response from inadequate target engagement [73]. Without such biomarkers, negative trial results are difficult to interpret: they may reflect a lack of efficacy, an insufficient dose for the individual patient, or enrollment of patients without a pre-existing redox imbalance. Fourth, as a narrative rather than a systematic review, this synthesis carries an inherent risk of selection bias in the identification and interpretation of studies, which a pre-registered systematic review with meta-analysis would mitigate. In addition, the immunological and anti-apoptotic mechanisms of NAC, which are hypothesized to contribute to its therapeutic effects, remain largely unexplored in clinical settings, as none of the trials reviewed assessed immune markers or apoptotic pathways as outcomes. An additional limitation of NAC is its relatively low systemic bioavailability and limited brain penetration. N-acetylcysteine amide, a more lipophilic derivative with enhanced membrane permeability and preclinical evidence of improved CNS penetration, may represent a future strategy to improve central delivery [37]. These limitations are precisely the gaps that the biomarker-enriched trial design outlined above aims to address, by selecting individuals with measurable redox imbalance, standardizing dose and duration, and incorporating mechanistic biomarkers as parallel endpoints.

## 6. Conclusions

NAC targets glutamatergic pathways related to NMDAR hypofunction, oxidative stress, and a possible low-grade neuroimmune activation, a network of mechanisms that converges on PVI dysfunction in SCZ. Negative symptoms and cognitive deficits, which remain largely refractory to APs, appear to respond favorably to NAC, whereas its benefit for positive symptoms is modest and inconsistent. Null findings in clinical-high-risk and treatment-resistant populations suggest that its efficacy may depend on a pre-existing redox imbalance. A biomarker-enriched trial evaluating NAC in patients with measurable antioxidant deficits could either establish it as a targeted therapy for a mechanistically defined subgroup or confirm that its modest effects in unselected populations do not justify routine use.

## Data Availability

No new data were created or analyzed in this study.

## Abbreviations

The following abbreviations are used in this manuscript:

AP	Antipsychotic
BACS	Brief Assessment of Cognition in Schizophrenia
CGI-S	Clinical Global Impressions-Severity
Cr	Chromium
dACC	Dorsal Anterior Cingulate Cortex
GABA	Gamma-Aminobutyric Acid
GSH	Glutathione
GSSG	Glutathione Disulfide
Hg	Mercury
•OH	Hydroxyl Radicals
IL-1ß	Interleukin-1 Beta
IL-6	Interleukin-6
mPFC	Medial Prefrontal Cortex
MRI	Magnetic Resonance Image
MRS	Magnetic Resonance Spectroscopy
NAC	N-Acetylcysteine
NMDAR	N-Methyl-d-Aspartate Receptor
NF-κB	Nuclear Factor Kappa-light-chain-enhancer of activated B cells
NO_2_	Nitrogen Dioxide
O_2_^−^	Superoxide
O_2_^2−^	Peroxide
PANSS	Positive and Negative Syndrome Scale
Pb	Lead
PFC	Prefrontal Cortex
PVIs	Parvalbumin-positive GABAergic Interneurons
RONS	Reactive Oxygen and Nitrogen Species
ROS	Reactive Oxygen Species
SCZ	Schizophrenia
SSD	Schizophrenia Spectrum Disorder
SZA	Schizoaffective Disorder
TNF-α	Tumor Necrosis Factor-Alpha
